# Effect of the Alterations in Contractility and Morphology Produced by Atrial Fibrillation on the Thrombosis Potential of the Left Atrial Appendage

**DOI:** 10.3389/fbioe.2021.586041

**Published:** 2021-02-26

**Authors:** Danila Vella, Alessandra Monteleone, Giulio Musotto, Giorgia Maria Bosi, Gaetano Burriesci

**Affiliations:** ^1^Bioengineering Unit, Ri. MED Foundation, Palermo, Italy; ^2^Department of Mechanical Engineering, University of Palermo, Palermo, Italy; ^3^UCL Mechanical Engineering, University College London, London, United Kingdom

**Keywords:** atrial fibrillation, computational fluid dynamic, cardiac wall motion, left atrial appendage, thromboembolic event

## Abstract

Atrial fibrillation (*AF*) is a common arrhythmia mainly affecting the elderly population, which can lead to serious complications such as stroke, ischaemic attack and vascular dementia. These problems are caused by thrombi which mostly originate in the left atrial appendage (*LAA*), a small muscular sac protruding from left atrium. The abnormal heart rhythm associated with *AF* results in alterations in the heart muscle contractions and in some reshaping of the cardiac chambers. This study aims to verify if and how these physiological changes can establish hemodynamic conditions in the *LAA* promoting thrombus formation, by means of computational fluid dynamic (CFD) analyses. In particular, sinus and fibrillation contractility was replicated by applying wall velocity/motion to models based on healthy and dilated idealized shapes of the left atrium with a common *LAA* morphology. The models were analyzed and compared in terms of shear strain rate (*SSR*) and vorticity, which are hemodynamic parameters directly associated with thrombogenicity. The study clearly indicates that the alterations in contractility and morphology associated with *AF* pathologies play a primary role in establishing hemodynamic conditions which promote higher incidence of ischaemic events, consistently with the clinical evidence. In particular, in the analyzed models, the impairment in contractility determined a decrease in SSR of about 50%, whilst the chamber pathological dilatation contributed to a 30% reduction, indicating increased risk of clot formation. The equivalent rigid wall model was characterized by SSR values about one order of magnitude smaller than in the contractile models, and substantially different vortical behavior, suggesting that analyses based on rigid chambers, although common in the literature, are inadequate to provide realistic results on the *LAA* hemodynamics.

## 1. Introduction

Atrial fibrillation (*AF*) is a pathological condition characterized by an irregular heart contraction pattern. *AF* can result in thromboembolic events, leading to serious complications such as stroke, ischaemic attack and vascular dementia. It is estimated that over 90% of the thrombi responsible for these pathologies originate in the left atrial appendage (*LAA*), a sac of muscle tissue protruding from the left atrium (*LA*) (Yaghi et al., 2015).

The *LAA* is highly variable in morphology and, in chronic *AF* (Lane et al., 2015), further changes are induced by remodeling. As a consequence, most of computational studies attempting to clarify the mechanisms promoting thromboembolism have focused on the hemodynamic impact of the different *LAA* anatomical shapes (Bosi et al., 2018; Masci et al., 2019). These studies have generally been based on patient-specific models, where the *LA* and *LAA* morphologies were reconstructed from medical imaging (Dedè et al., 2019). However, the large anatomical variability and the influence of several related parameters reduce the ability to generalize the results from these studies, leading to lack of agreement in their interpretation (Di Biase et al., 2012; Korhonen et al., 2015). Moreover, reference morphologies are often reconstructed at a specific phase of the cardiac cycle, and do not replicate the geometric changes occurring during normal function. This work investigates the effect on the local hydrodynamics of the typical changes in contractility and shape experienced in the *LA* and *LAA* as effect of *AF* (Farese et al., 2019). In fact, *AF* is associated with loss of contractility in the atrial muscles, resulting in abnormal dynamics and expansion of the atrial chambers (Sanfilippo et al., 1990; Mori et al., 2011; Nattel and Harada, 2014). Moreover, if chronic, it commonly determines an expansion of the *LA* and *LAA* volumes (Schwartzman et al., 2003; Lacomis et al., 2007). To take into account these morphological changes, two idealized models of the *LA* and *LAA*, representative of healthy and *AF* patients were created from CT images of healthy and pathological patients' groups (Lacomis et al., 2007). These idealized models of reduced complexity were preferred, as they allow to focus on the contribution from each individual parameter (i.e., contractility and reshaping), providing a clear indication on the fluid dynamic changes that they introduce in the system, allowing easier generalization of the findings (Dedè et al., 2019).

## 2. Methods

In order to verify the contribution from the alterations produced by *AF*, in terms of contractility and morphology, four scenarios were simulated; three based on a morphology representative of healthy conditions, where the wall (i) is rigid, (ii) replicates contractions typical of *SR*, and (iii) replicates contractions typical of *AF*; and one based on a pathologically enlarged morphology, undergoing wall contractions typical of AF. The four scenarios were designed to analyse the effect on the hemodynamics of two main factors associated with AF, the impairment in the contraction and the progressive enlargement of the *LA*/*LAA* chambers. The contraction of the models was replicated by controlling the instantaneous flowrate through the mitral valve (by adjusting the pressure difference between the pulmonary veins inlet and the mitral outlet), the wall velocity of the atrium and the wall motion of the appendage. The chambers' enlargement was considered by analyzing two idealized morphologies of the atrium and appendage, whose volumes were set to reproduce the average dimensions of the *LA* and *LAA* reported for the healthy and *AF* cases. The results obtained were compared in terms of Shear Strain Rate (SSR) and vortex structures, which are parameters related to the thrombogenic potential (Kim et al., 2012; Otani et al., 2016). Four cardiac cycles were simulated for each scenario, assuming a heart rate of 70 beats/min, using the commercial software Ansys CFX 19.2. The workflow followed in this study can be described by the following main steps:

- **Step 1** Design of two *LA* and *LAA* idealized models, describing the average healthy and pathological morphologies, respectively;
- **Step 2** Definition of Boundary Conditions: inlet (at the pulmonary veins) and outlet (at the mitral valve orifice) *Pressures* in healthy and pathological conditions;
- **Step 3** Definition of contraction functions for the *LA* and *LAA* walls, in healthy and pathological conditions (in particular, *Wall Velocity* conditions were applied to the *LA*, whilst a higher degree of accuracy was preferred for the *LAA* portion, by imposing *Wall Motion*);
- **Step 4** Setting up of four scenarios (see Table 1):
  - **Rigid** - represents the healthy morphology in absence of contraction,
  - **Healthy** - represents the healthy morphology with sinus rhythm (*SR*) contraction pattern,
  - **Hybrid** - represents the healthy morphology with *AF* contraction pattern,
  - **Pathological** - represents the pathological morphology with *AF* contraction pattern.

**Table 1:** Combinations of geometries and functions defining the four scenarios.

	**Geometry**	**Pressure**	**AWM**	**AWV**
Rigid	H	H	–	–
Healthy	H	H	H	H
Hybrid	H	P	P	P
Pathological	P	P	P	P

*H, healthy; P, pathological; AWM, Appendage Wall Motion; AWV, Atrium Wall Velocity*.

In particular, in the *Rigid* model the flow is established by only applying the physiological pressure difference between the inlet pulmonary veins and the mitral outlet, similarly to a number of numerical studies in the literature (Bosi et al., 2018). The *Healthy* model incorporates the physiological contractions reported for the atrium and for the appendage, thus providing a representation of normal operating conditions. The *Hybrid* model applies pressure differences and contractions descriptive of *AF* to the same morphology, introducing the isolated effect of the loss in contractility. Comparison of these three models clarifies the role of a physiological contraction, and of the implication of neglecting the wall motion. Finally, the *Pathological* model includes in the *Hybrid* model the morphological alterations typically associated with chronic fibrillation, thus allowing to isolate the effect of the cardiac chambers' expansion observed in patients suffering from AF.

### 2.1. The Idealized Geometries

Two idealized geometries of the *LA* were defined, which are representative of the healthy adult population in *SR* conditions and of the adult population affected by *AF*. The two geometries were obtained from clinical measurements on *SR* and *AF* populations reported in the literature (Lacomis et al., 2007).

Each model comprises two main parts, the appendage and the main atrial chamber; which were built separately and then integrated into a single structure using the Computer Aided Design (CAD) software, Rhinoceros 6.0 (further details are described in Supplementary Material 1).

**Figure 1:**
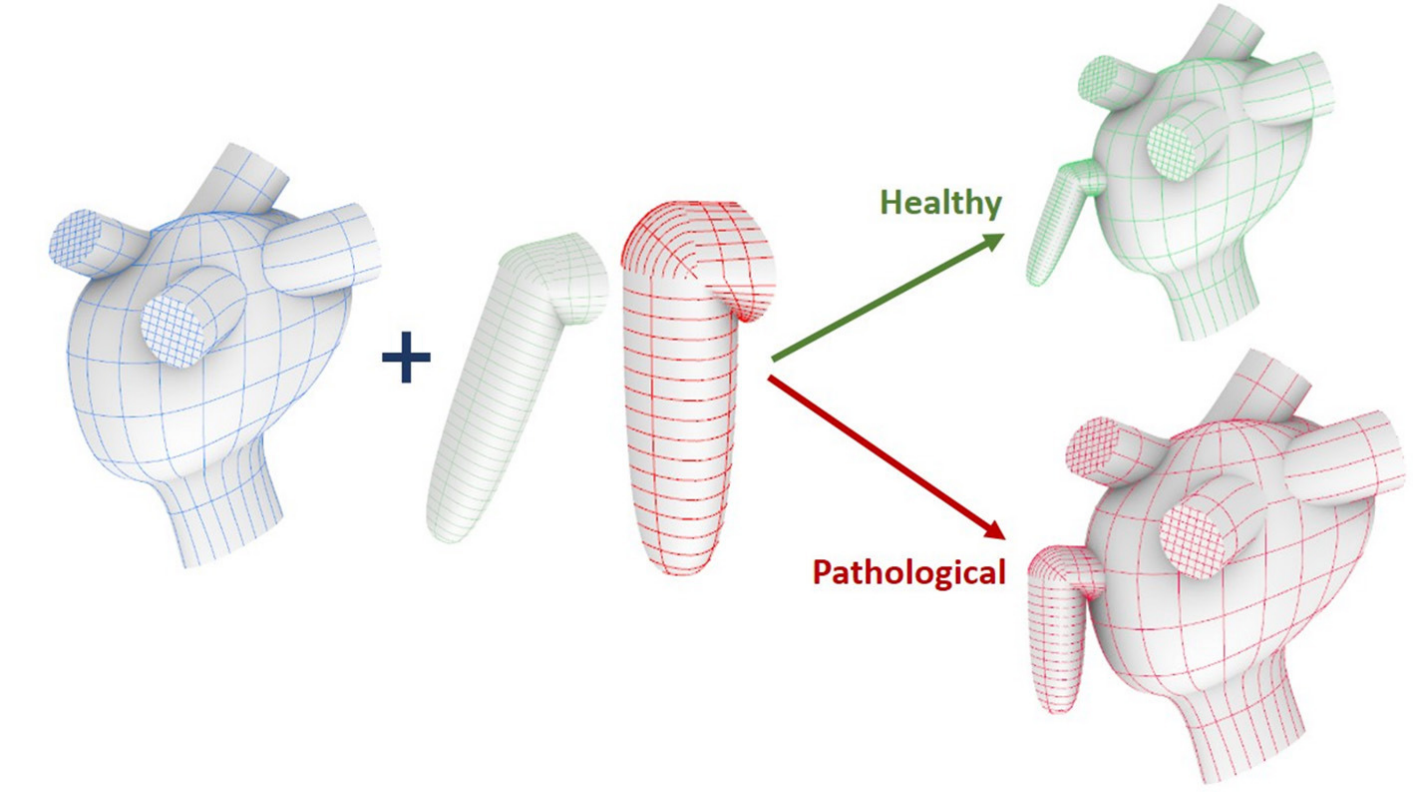
Construction workflow of the atrium model. From top to bottom: main ideal shape, pulmonary veins and mitral valve.

Icem CFD was used for meshing the two parts. The mesh was made of tetrahedral and prism elements with 5 layers of prism elements along the wall. In order to maintain the same element density in the *Pathological* and *Healthy* models, whose computational domains have different volumes (see Figure 1), the final meshes contain a total of 4548050 and 2888185 elements, respectively (Bosi et al., 2018).

#### 2.1.1. Appendage Model

The appendage morphology is highly variable from patient to patient, making difficult to classify the existing shapes according to objective criteria. Hence, the most commonly adopted classification is based on a qualitative evaluation, where the appendage is associated to specific classes inspired by common objects with resembling shape: Chicken Wing, Cactus, Windsock, and Cauliflower (Nedios et al., 2014; Korhonen et al., 2015). In this study, the ideal geometry was based on the Chicken Wing morphology, which is the most common (Di Biase et al., 2012) and the easiest to create from the available clinical measurements. As a result, the geometry of the appendage model comprises two portions: the proximal region and the distal region. The former departs from the main atrial chamber and consists of a tubular duct which connects the atrial orifice to the distal region. The distal region consists of a semi-ellipsoidal shape, which starts from the proximal region and extends at an angle, terminating with a tip. Both, the orifice of the proximal part and the orifice of the distal part, have elliptical cross sections. The model is based on seven parameters: (i) maximum proximal orifice diameter; (ii) minimum proximal orifice diameter; (iii) length of the proximal part; (iv) maximum distal orifice diameter; (v) minimum distal orifice diameter; (vi) length of the distal part; and (vii) the angle α of proximal-distal joint (see Table 2 and further details in Supplementary Material 1.2).

**Table 2:** Parameters defining the appendage idealized models.

	**Healthy**		**Pathological**	
	**LAAp**	**LAAd**	**LAAp**	**LAAd**
Diameter max [mm]	19	13	22	18
Diameter min [mm]	10	10	13	14
Length [mm]	5	31	6	34
Angle α	114°		92°	

*LAAd, appendage distal part; LAAp, appendage proximal part; MV, mitral valve; LSPV, left superior pulmonary vein*.

#### 2.1.2. Atrium Model

The atrium model comprises three main sections: main chamber, pulmonary veins and mitral valve. The main chamber was designed using an average shape obtained from a population study by (Varela et al., 2017). The pulmonary veins access portions were created and inserted based on clinical measurements of the orifice diameters and the relative distances by (Schwartzman et al., 2003); and the mitral valve was designed as a funnel shape tube with an elliptical orifice, positioned at the lower portion of the main atrial chamber (Lim et al., 2005; Bloodworth et al., 2017) (further detail are provided in Supplementary Material 1.1). The model was designed to obtain a generalized shape able to represent a standard atrium, then scaled to the volume reported from clinical measurements of the healthy and pathological subject groups (Lacomis et al., 2007). These two volume values (85.5 and 117.5 ml, respectively) were computed as average between the maximum and minimum atrial volumes measured during the cardiac cycle.

### 2.2. Appendage Wall Motion

**Figure 2:**
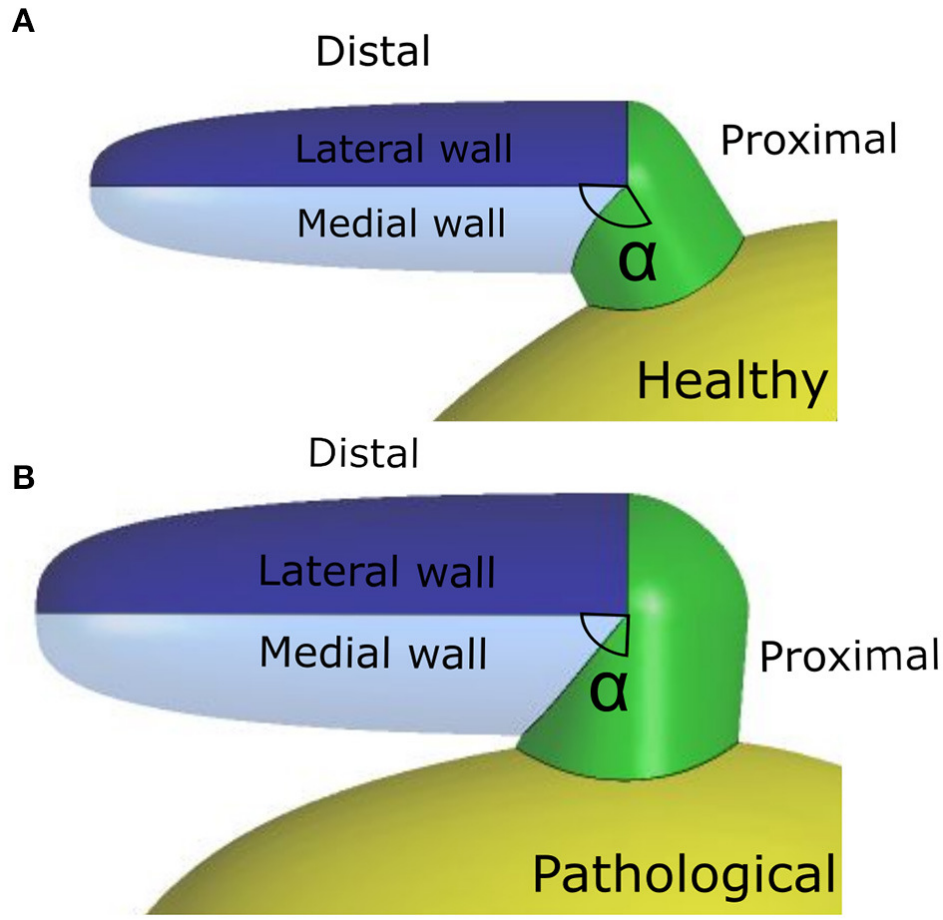
**(A)** *Healthy* (H) geometry; **(B)** *Pathological* (P) geometry.

**Figure 3:**
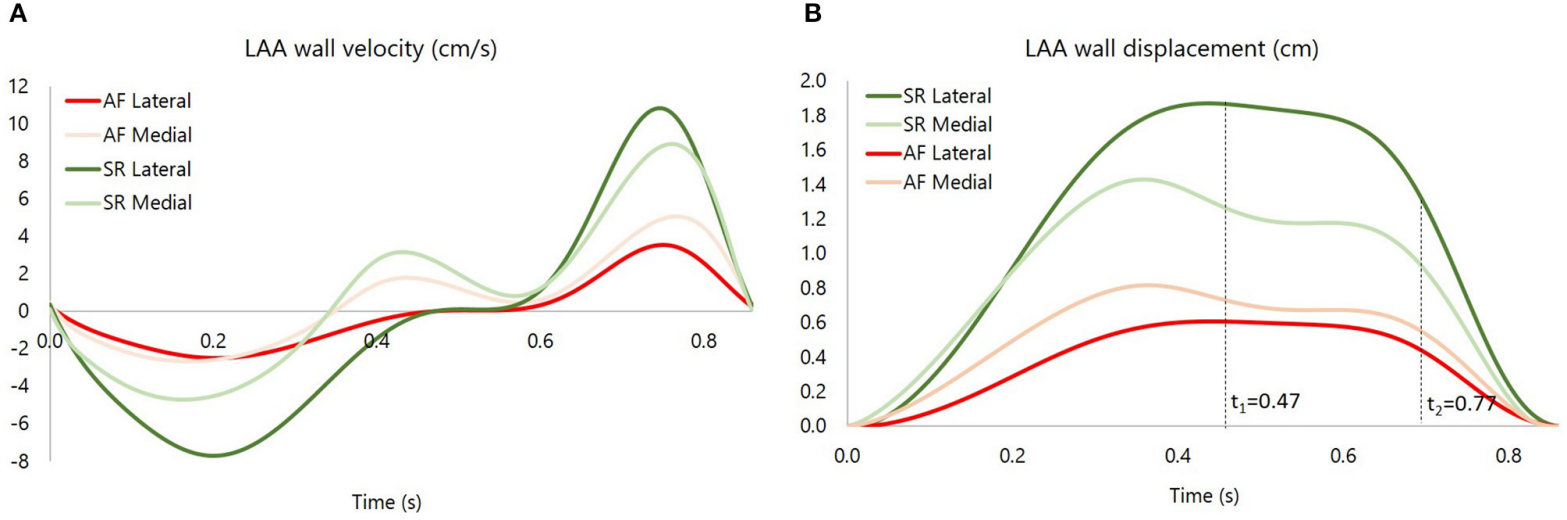
**(A)** medial and lateral wall velocity of the appendage for the *Pathological* (P) case and the *Healthy* (H) case. **(B)** medial and lateral wall displacement, respectively, *s*_*M*_ and *s*_*L*_, for the *Pathological* (P) case and the *Healthy* (H) case. The instants *t*_1_ = 0.47 s and *t*_2_ = 0.77 s are showed, corresponding, respectively, to E wave and A wave.

As discussed in section 2.1, the *LAA* idealized structure is composed by a proximal region and a distal region. This part is in turn partitioned into the medial wall, facing the main atrium chamber, and the lateral wall, further from the *LA* (see Figure 2). In the implemented model, the contraction of the appendage was only applied to the distal part, for which experimental data are available in the literature (Farese et al., 2019), and was defined imposing two different displacement functions for the lateral and medial wall, *F*_*L*_(*t*) and *F*_*M*_(*t*), respectively. These functions are the result of mathematical transformations based on two main displacement curves, *s*_*L*_(*t*) and *s*_*M*_(*t*), obtained by integrating over time the velocities of the two appendage walls. The function magnitudes were linearly varied from the center-line of each wall section to the line where these join, in order to have a smooth transition in the movement enforced in the two sections. A detailed description of the two functions, *F*_*L*_(*t*) and *F*_*M*_(*t*), is provided in Supplementary Material 2. In particular, the wall velocities of a representative patient in *SR* were extracted from Tissue Doppler echocardiography data reported in the literature (Farese et al., 2019). The curve amplitudes and inflection points were then adjusted to fit the mean values of the *Healthy* and *Pathological* populations (Figure 3, left panel). Interestingly, the direction of the lateral and medial wall motion during the cardiac cycle reverts in the *Pathological* model, compared to *Healthy* conditions (Figure 3, right panel) (Farese et al., 2019). This is due to the fact that under healthy conditions the *LAA* experiences an active contraction, while during *AF* it mostly expands passively, under the effect of the atrial pressure.

### 2.3. Atrium Wall Velocity

To take into account the atrial contraction without increasing substantially the model complexity, the velocity magnitude was set to reproduce the volume variations reported for *SR* and *AF* patients (Lacomis et al., 2007), normalizing with respect to the wall area and differentiating with respect to the time. The motion was modeled by applying to the fluid a velocity normal to the atrial wall. Details on the determination of the wall velocities are provided in (Supplementary Material 3).

### 2.4. Pressure

**Figure 4:**
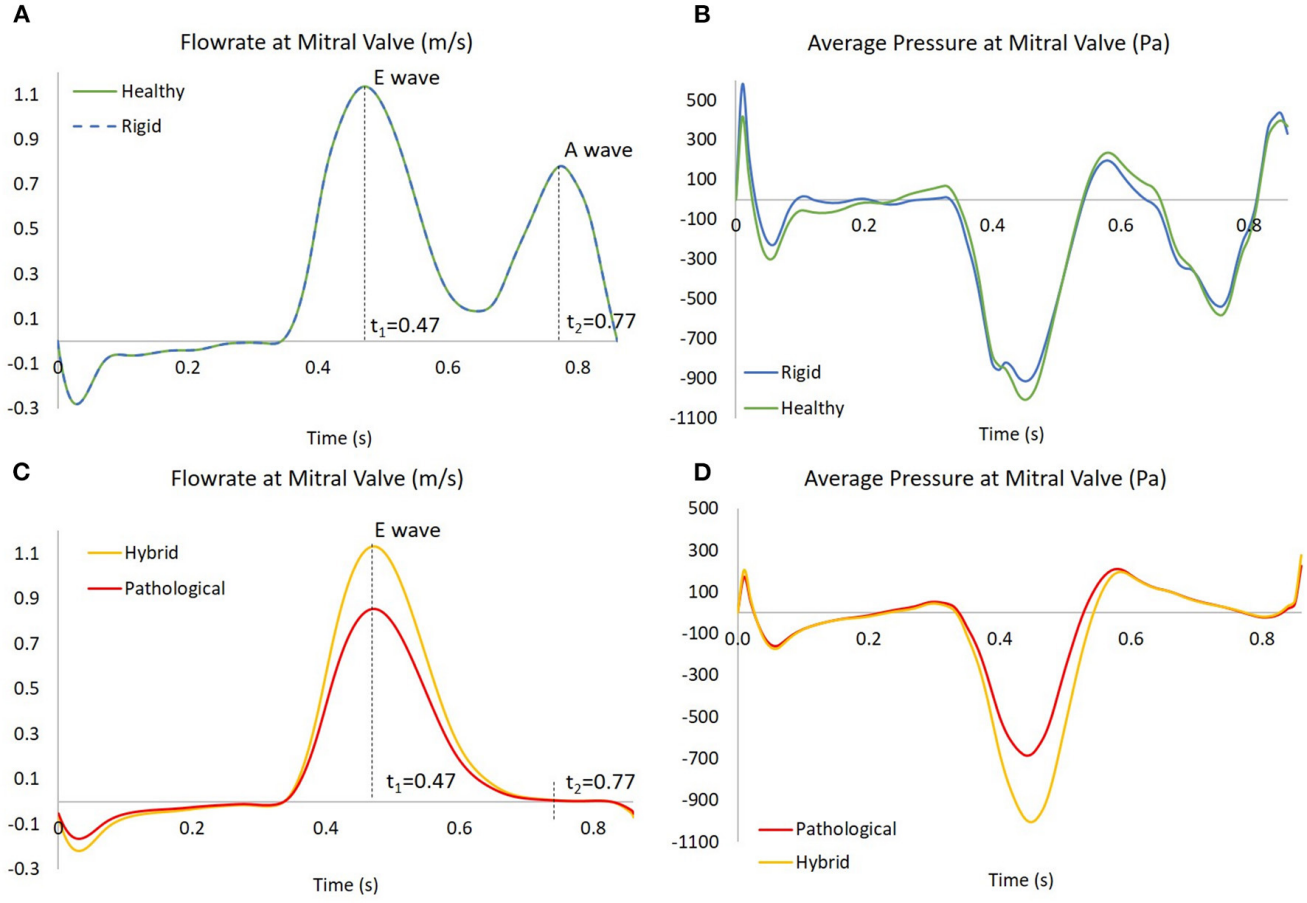
In **(A,C)**, the flowrates at mitral valve for the four scenarios; the instants *t*_1_ = 0.47 s and *t*_2_ = 0.77 s are showed, corresponding to E wave and A wave, respectively. In **(B,D)**, the pressures at mitral valve for the four scenarios, obtained as results of four simulations based on the flowrate in the left panel.

To simulate the blood flow into the atrium, pressure was imposed at both, the inlet of the pulmonary veins and the outlet of the mitral valve. For all cases, pressure at the pulmonary veins was set to a constant zero value. A uniformly distributed pressure was imposed at the mitral valve outlet, to produce a flowrate representative of *SR* and *AF* cases, as shown in Figure 4 (more details on the setting of the pressure boundary condition are provided in Supplementary Material 4).

The flowrate was derived from the international standard ISO 5840-1:2015, to represent the flow across the mitral valve for a generic healthy subject (Bosi et al., 2018).

## 3. Results and Discussion

Two hemodynamic parameters were investigated in the four models: SSR and vortex core regions. The SSR is a measurement of the flow velocity related to crossed space dimensions, and is linked to the rheological response as well as to thrombogenicity of blood (Cadroy et al., 1989; Ducci et al., 2016; Casa and Ku, 2017; Bosi et al., 2018). Although the velocity is the most used parameter to evaluate hemodynamics (Dedè et al., 2019; Jia et al., 2019; Masci et al., 2019), SSR is better descriptive of the flow features in a context where different global velocities are applied to the study chamber, due to the alternative *LAA* motion modes analyzed in this study. For the computation of the vortex structures, the Q-criterion was employed. Q-criterion is a measure based on the second invariant of the velocity gradient tensor, widely used to evaluate stagnation regions and blood washing, which are directly related to clot formation (Chnafa et al., 2014; Seo et al., 2014; Vedula et al., 2015; Otani et al., 2016; Dedè et al., 2019; Masci et al., 2019). Specifically, a *Q* function is calculated as

(1) $$\begin{array}{l} Q=\frac{1}{2}\left(\Omega^{2}-S^{2}\right) \end{array}$$

**Ω** and ***S*** are the skew-symmetric and the symmetric components of the velocity gradient tensor, respectively.

**Figure 5:**
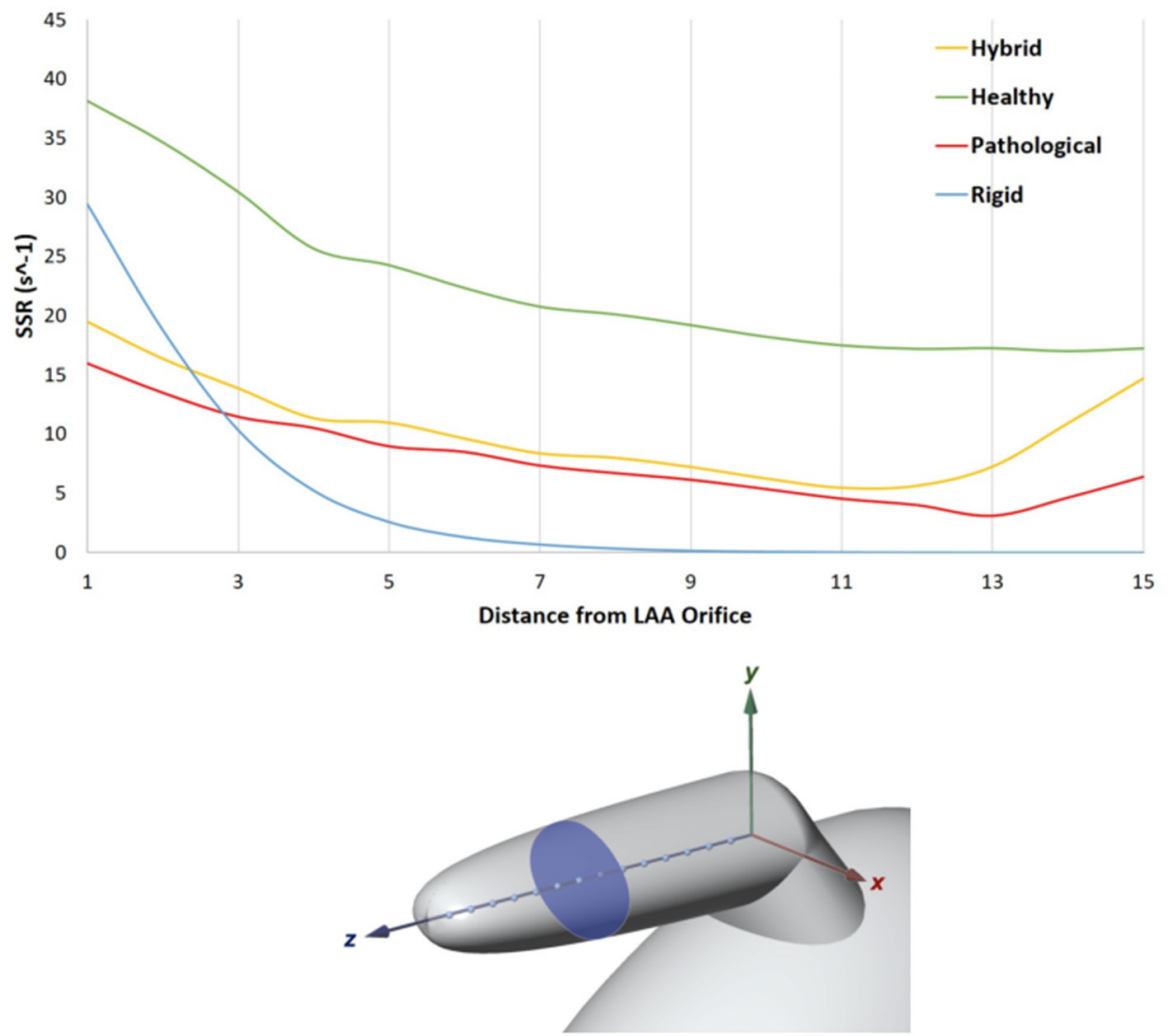
In the top panel SSR along the *LAA* axis, from the orifice to the tip. LAA axis at the fourth SSR peak of the cardiac cycle (*t* = 3.36 s, *Rigid* case).

The contour maps obtained for all models during the cardiac cycle can be seen in the videos *Rigid, Healthy, Hybrid*, and *Pathological*, included as part of the Supplementary Materials. In the description below, a local Cartesian system will be used as reference, with the origin positioned at the intersection between the axes of the distal and proximal *LAA* portions; the x-axis in the plane separating the lateral and medial walls; the y-axis directed along the normal to this plane, directed toward the lateral walls; and the z-axis aligned with the axis of the distal portion and directed toward the tip of the *LAA* (see Figure 5, bottom panel).

An analysis of the SSR along the *LAA* axis was performed at the peak flowrate of the fourth cardiac cycle, (*t* = 3.36 s in the analysis). The *LAA* z-axis of the four models was divided into *N* = 15 equally spaced points (Figure 5, bottom panel) and the SSR average value was computed on the transversal cross sections passing through each of these points (one of the transversal cross section is highlighted in Figure 5, bottom panel). The diagram in Figure 5 (top panel) represents the SSR values obtained for all cases (the maximum and the average values for the 15 sections are reported in Table 3 last column).

**Table 3:** SSR maximum values (average values into brackets) for the four scenarios evaluated in sagittal (S) section planes (Superior, Central, and Inferior) and Transversal planes.

	**Superior S [s^**−1**^]**	**Central S [s^**−1**^]**	**Inferior S [s^**−1**^]**	**Transversal [s^**−1**^]**
Rigid	5.09 (1.32)	0.23 (0.07)	0.02 (0.005)	29.38 (4.59)
Healthy	35.49 (18.11)	22.53 (11.87)	17.84 (10.56)	38.14 (22.64)
Hybrid	16.34 (8.24)	9.58 (5.29)	6.49 (4.41)	19.49 (10.35)
Pathological	11.53 (5.85)	6.79 (3.61)	4.40 (2.74)	15.98 (7.81)

In the simulation with healthy geometry and without contraction of the appendage (blue line in the figure), the mean cross sectional SSR quickly decreases to zero value from the orifice to the tip of the *LAA*, reducing of over one order of magnitude in the first proximal third of the axis. It should be highlighted that, in real conditions, sustained SSR values below 10 s^−1^ would result in substantial increase in blood viscosity (Chien, 1981; Cho et al., 2014; Ducci et al., 2016). This non-Newtonian behavior, neglected in this study, would further contribute to generate blood stagnation in the appendage. This condition, which typically promotes clot formation, is prevented in the *Healthy* model, where the *LAA* motion is applied (green line). In this case, results indicate a much more favorable scenario, with mean cross sectional SSR values maintained well above 10 s^−1^ up to the distal tip of the appendage. This highlights the crucial importance of including the wall motion when simulating the phenomenon. In the *Pathological* case (red line in the figure), as a result of the irregular and reduced volume contraction, SSR values in most of the distal region are below 10 s^−1^. In the *Hybrid* case (yellow line), characterized by healthy geometry and *AF* contraction, a very similar trend as in the *Pathological* model is observed, except for the tip region, where the SSR values increase. This effect may be due to the different size of the chamber.

**Figure 6:**
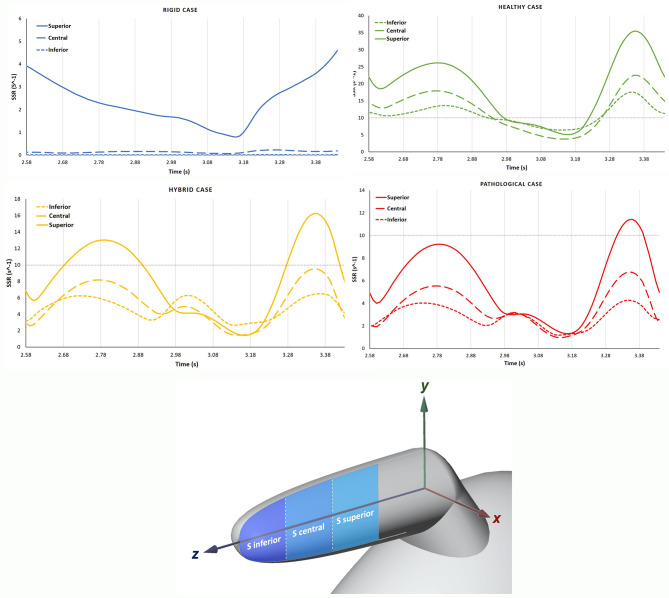
In the **top** panel, the SSR in the S sections are compared among the different cases, *Rigid, Healthy, Pathological* and *Hybrid*. In the **bottom** panel, the distal part with sagittal (S) sections: Inferior, Central and Superior.

The SSR was also assessed on three sagittal (S) sections (see Figure 6, bottom panel) of the appendage distal part, named Inferior, Central and Superior. These S sections were selected close to the tip, because this is the region where the development of thrombi is most likely to occur. Each section has the same length along *z* and normal aligned with the x direction. The average SSR computed for all the S sections during the fourth cardiac cycle are represented in Figure 6 top panel. The graphs show marked differences between the average SSR values measured in the four analyzed cases, as summarized in Table 3 (the first three columns for the S sections). For the *Rigid* model, the average SSR varies in the range 0 ÷ 5 s^−1^ and keeps always well below 10 s^−1^. In the *Healthy* case, the SSR ranges between 0 ÷ 35 s^−1^ and it reduces below 10 s^−1^ only for a short period of about 0.3 s, during the ventricular diastole. In the *Hybrid* model, it varies in the range 0 ÷ 16 s^−1^ and it is below 10 s^−1^ during most of the cardiac cycle. In the *Pathological* case it varies in the range 0 ÷ 11 s^−1^ and it keeps below 10 s^−1^ for nearly the entire cycle. For all cases, the SSR values gradually decrease along the z-axis, moving toward the tip, from the superior section to the inferior section (see Figure 6 top panel).

**Figure 7:**
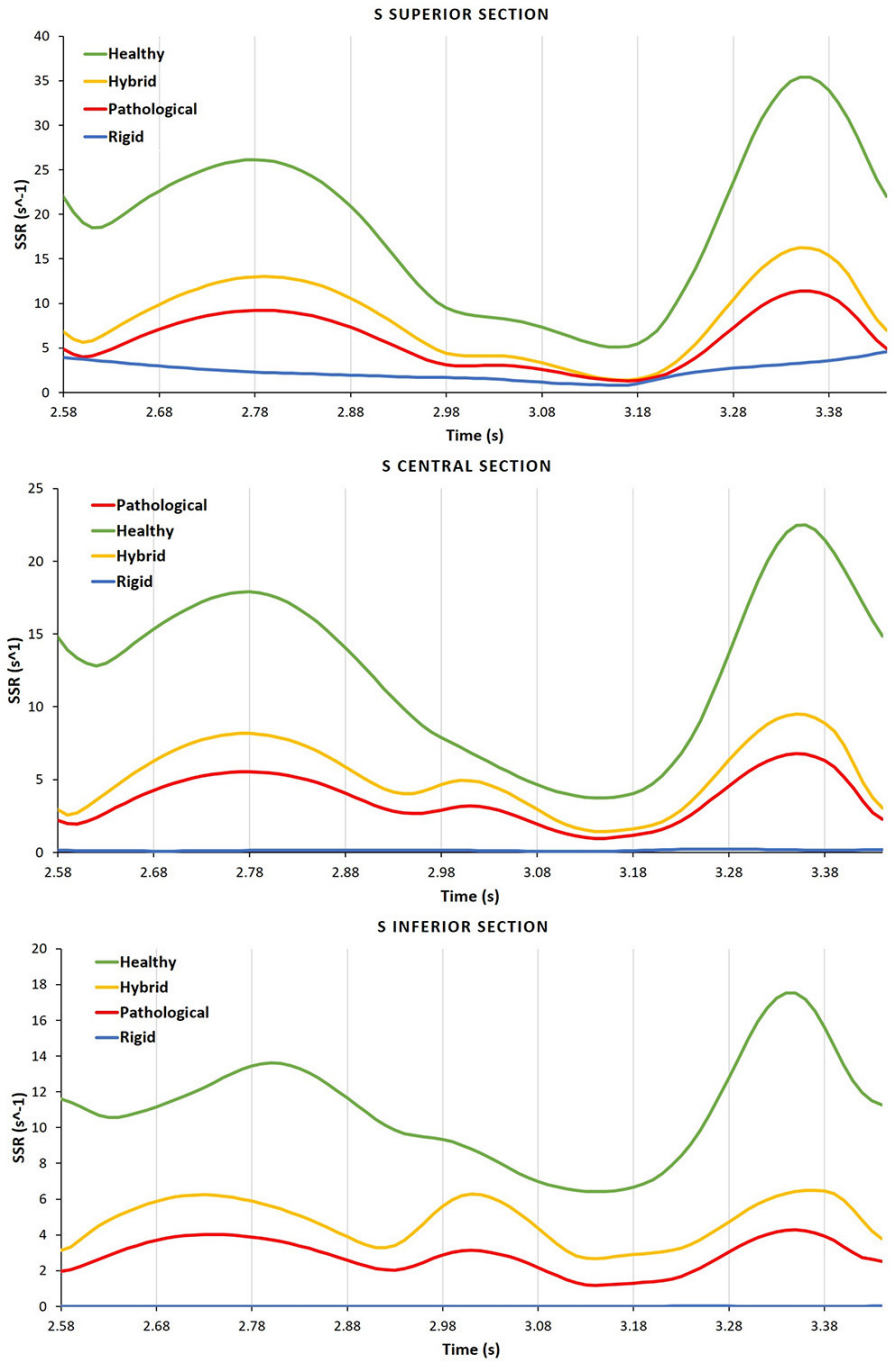
For each case the SSR is analyzed in the different S sections, Inferior, Central, and Superior.

Figure 7 compares the SSR trends for the four scenarios. The difference between the *Healthy* and *Hybrid* curves (green and yellow lines) indicates that the alteration in contractility due to *AF* contributes to a substantial decrease in SSR, which in our simulations is of about 50%. The *Pathological* curve (red line), which also models the volume expansion associated with chronic *AF* further exacerbates the reduction in SSR, producing a decrease of about 30% from the *Hybrid* model. In the *Rigid* model the SSR follows a different trend, keeping one order of magnitude lower than other cases. This indicates that the rigid chamber assumption, although widely used in literature, is inadequate to investigate *LAA* hemodynamics.

**Figure 8:**
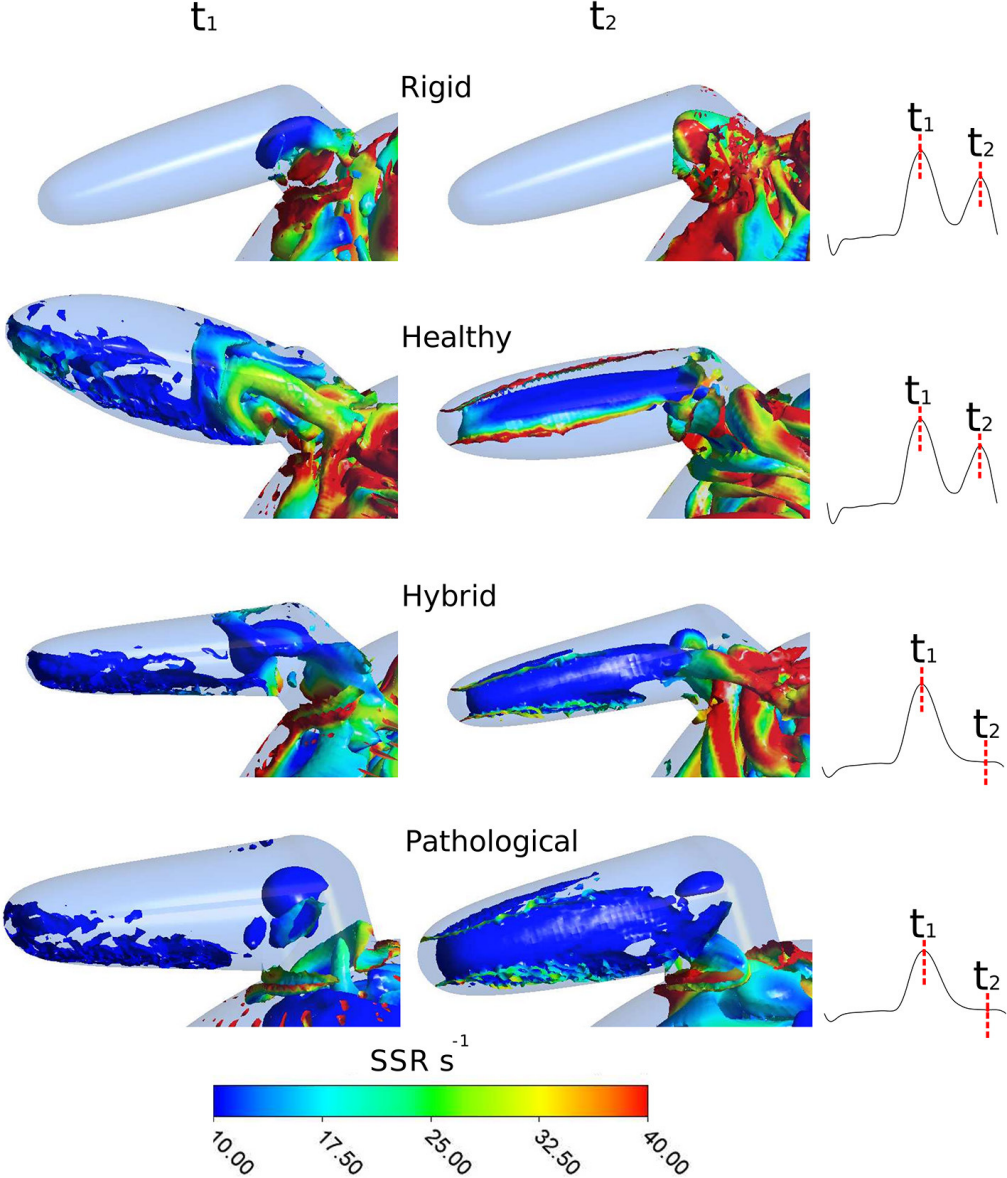
Instantaneous vortex structures within *LAA* colored according to the SSR parameter, at E wave peak (corresponding to *t*_1_) and at A wave peak (corresponding to *t*_2_) of the fourth cardiac cycle. To visually appreciate the extent of the regions prone to thrombus formation, the SSR scale starts from 10 s^−1^. First row: *Rigid*; second row: *Healthy*; third row: *Hybrid*; fourth row: *Pathological*.

As mentioned above, the identification of vortex core regions is a useful indicator of the complex pattern of blood flow occurring in the appendage. Figure 8 represents, for each model, the instantaneous vortex structures within the *LAA* at the early ventricular diastolic peak *E* (corresponding to time *t*_1_ = 0.47 s in Figure 4, left panel), and at the atrial systole peak *A* (corresponding to time *t*_2_ = 0.77 s in Figure 4, left panel) of the fourth cardiac cycle. Vortex structures are visualized as iso-contours of *Q*, selecting a suitable positive value. The color of the contours indicates the SSR computed over the surface. For the *Rigid* model (first row in Figure 8), vortexes are confined in the proximal *LAA* region and do not expand along the appendage distal direction. Vortexes with higher SSR values are observed at time *t*_2_. When the *LAA* contraction is included for the *Healthy* case (second row in Figure 8), the vortex core regions expand into the appendage, reaching its tip with SSR value higher than 10 s^−1^. This indicates a better blood washout. In particular, at time *t*_1_, the *LAA* reaches the maximum volume (see dark and light green curves in Figure 3, right panel) and a well-structured vortex is clearly visible. At this instant the SSR ranges from 25 to 35 s^−1^ in the early distal region and shows a value of about 20 s^−1^ in the region of the appendage tip. At time *t*_2_ (left atrial contraction phase) blood flows from the appendage to the main atrial chamber; destroying the vortexes observed previously and keeping high SSR values at walls. These events indicate a reduced blood stagnation and consequently lower risk of clot formation. When the *AF* contraction is imposed (in the *Hybrid* and the *Pathological* cases), the *LAA* volume change between *t*_1_ and *t*_2_ is reduced compared to the model with *SR* contraction (*Healthy*) and the wall motion is inverted, with the medial (inferior) *LAA* wall displacement larger than the lateral one (superior) (see light red and dark red lines in Figure 3, right panel). Moreover, the A wave associated with the atrial systole is not present (Bosi et al., 2018). For the *Hybrid* model (third row in the figure), although the vortex structures are similar to those obtained for the *Healthy* case, the SSR values are lower. In the *Pathological* model, at time *t*_1_ (fourth row in Figure 8), the vortexes in the appendage appear less pronounced (some structure with very low SSR values can be observed). At time *t*_2_, vortexes still have SSR values lower than 10 s^−1^ in almost the entire appendage volume, thus implying a worse blood washout compared to the *Healthy* case. Of course, the presented results are based on clinical AF data on patients with more severe symptomatology and idealized anatomies. Large variability is to be expected, depending on patient specific morphologies and on the severity and degree of persistence of the condition.

These results further confirm the key role of the appendage wall motion in producing safe or potentially thrombogenic hemodynamic conditions. This suggests that features influencing the fluid dynamics in the *LAA*, such as the contractility and shape, may enhance the prediction of ischaemic events. In fact, the most used criteria currently adopted for *AF* patients stratification, such as ChadS2 and CHA2DS2-VASc (Lip et al., 2010; Kimura et al., 2013), assigns points according to existing diseases (e.g., diabetes and hypertension), age and gender, without taking into account any direct effect of the hemodynamics. In this context, models like the one presented in this paper, based on the analysis of SSR and vorticity in different situations, could find relevant applications in the clinical practice, providing more accurate predictions of the ischaemic event risk, especially for low CHA2DS2-VASc scores (e.g., < 2). This will offer a more effective tool in support of the most adequate therapeutic selection. Currently, the available therapies to minimize the ischaemic risk in *AF* patients include oral anticoagulation, surgical *LAA* exclusion and percutaneous *LAA* occlusion devices (Gan et al., 2014). However, none of the current solutions is free from potentially major complications; oral anticoagulation is associated to haemorrhagic risk (Onalan et al., 2005; Hylek et al., 2008), surgical *LAA* exclusion exposes patients to invasive surgery (Ailawadi et al., 2011), and percutaneous devices can be associated with vascular complications, air-embolic events, and peri-device leaks (Lam et al., 2013). Hence, a more informed selection of the most suitable therapy for a specific *AF* patient, supported by a better understanding of the phenomena responsible for ischaemic complications, may assume a key role in next generation clinical strategies.

## 4. Conclusion

This study allowed to investigate the effect of the changes in contractility and shape occurring in *AF* patients on the local hemodynamics that establishes into the *LAA*. The analysis reveals the essential role of the *LAA* wall contractility on the factors that may promote the formation of thrombus and consequent ischaemic complications. The described model is based on a number of assumptions, such as the laminar Newtonian description of blood and the simplified anatomical geometry. New models shall be implemented to overcome these approximations. Despite these limitations, the study indicates that modeling the morphological and contractile alterations typically caused by *AF* pathologies allows to replicate flow conditions which justify the high incidence of observed ischaemic events. The *LAA* impaired contraction resulted to be the main cause in flow dynamics worsening. However, also the enlargement of the cardiac chambers associated with chronic *AF* appeared to have a relevant effect. This confirms that the *LAA* morphological class may have a role in determining thrombogenic hemodynamic conditions in pathological patients. However, the investigation of the morphological effects cannot neglect the modeling of the *LAA* contractility to provide an acceptable analysis of the flow that establishes in the region. The proposed model lays the foundation for developing new computational studies to better understand the *AF* pathology and the factors that contribute to major complications. Moreover, the availability in literature of clinical data from Tissue Doppler echocardiography, describing the *LAA* movement associated to other pathologies, leads to new potential applications of the model. In fact, thanks to its simplified geometry, the presented model is suitable to reproduce other conditions associated with impaired atrial contractility and increased risk of thromboembolic events, such as paroxysmal supraventricular tachycardia and atrial fibrosis (Kamel et al., 2013; Sohns and Marrouche, 2020).

## Data Availability Statement

The original contributions presented in the study are included in the article/Supplementary Material, further inquiries can be directed to the corresponding author/s.

## Author Contributions

All authors was fully involved in the study and has contributed significantly to the submitted work, in terms of conception and design of the study, analysis and interpretation of the results and critical review of the manuscript.

## Conflict of Interest

The authors declare that the research was conducted in the absence of any commercial or financial relationships that could be construed as a potential conflict of interest.

## Supplementary Material

Supplementary Video 1 Rigid. SSR during a cardiac cycle (0.86 s) in the sagittal plane of the appendage body.

Supplementary Video 2 Healthy. Appendage wall motion in the healthy case during a cardiac cycle (0.86 s), the sagittal section is colored according SSR values.

Supplementary Video 3 Hybrid. Appendage wallmotion in the hybrid case during a cardiac cycle (0.86 s), the sagittal section is colored according SSR values.

Supplementary Video 4 Pathological. Appendage wall motion in the pathological case during a cardiac cycle (0.86 s), the sagittal section is colored according SSR values.
