# Supplementary material for: Effect of the Alterations in Contractility and Morphology Produced by Atrial Fibrillation on the Thrombosis Potential of the Left Atrial Appendage
Source: Front Bioeng Biotechnol. 2021 Feb 26;9:586041. doi: 10.3389/fbioe.2021.586041 (PMC7952649; doi:10.3389/fbioe.2021.586041)
Supplement: Supplementary file 5 [file Data_Sheet_1.PDF]

Supplementary Material

1 IDEALIZED MODELS

1.1 Atrium model

Below, the procedure used to create the atrium model is described in detail. The model comprises three portions: ideal main atrial chamber; pulmonary veins and mitral valve (see Figure S1). The model was designed to represent the shape of a standard atrium. The volume of the model can be changed according clinical measures in order to represent a specific case or a specific population.

Ideal main atrial chamber

The standard shape of the atrium was obtained from the study by Varela et al. (2017), providing the maps of the left atrium geometry obtained from magnetic resonance images of 144 AF patients. The average shape of all atrial chamber surfaces was selected to define the ideal main atrial chamber.

Pulmonary veins

To build the pulmonary veins, firstly the ideal main atrial chamber was scaled according the atrium average dimensions (x,y,z), derived by a population study of AF patients (Schwartzman et al., 2003). Then, the veins were created and inserted into the atrium, according to the clinical measurements of the same population, as summarised in Table S1. The values used to define the veins geometries were (i) dimensions of the orifice at the access of each vein into the atrial chamber; (ii) mutual distances between the access orifices; and (iii) distances between each access orifice and the mitral valve orifice (Schwartzman et al., 2003).

Mitral valve

The mitral valve was designed as a tubular shape set at the atrium base, extending normal to the surface and reducing in diameter with a funnel shape (Lim et al., 2005; Bloodworth et al., 2017).

The atrial geometry obtained as described above, based on data from AF patients, was scaled in volume to represent healthy conditions. In fact, although it is clear from the literature that AF causes a volume enlargement, there are no evidence that significant shape variations occur (Sanfilippo et al., 1990; Nattel and Harada, 2014; Mori et al., 2011).

1.2 Appendage model

The geometry of the appendage includes two portions: the proximal region and the distal region. The former is adjacent to atrium and consists of a tubular shape, which starts from the atrial orifice and extends to the distal part with a specific angle. The distal region consists of a tubular shape of ellipsoidal shape, which starts from the proximal part and terminates with a tip Budge et al. (2008). The model is based on seven parameters: maximum orifice diameter ($D_{p,max}$), minimum orifice diameter ($D_{p,min}$) and length of the proximal part (L_p); maximum orifice diameter ($D_{d,max}$), minimum orifice diameter ($D_{d,min}$) and length of the distal part (L_d) and the angle of connection (α). The parameters used are reported in Table 2 in the main text, both for Healthy case and Pathological case, as described in Lacomis et al. (2007). The steps followed for the construction of the appendages are described in Fig. S2. The appendages were attached to the atrial chamber at a distance from the mitral valve double than the distance to the left superior pulmonary vein and orienting the tip towards the base, as described in Li et al. (2015). These geometrical features were adopted for both, the *Healthy* and *Pathological*, are summarized in Table S2.

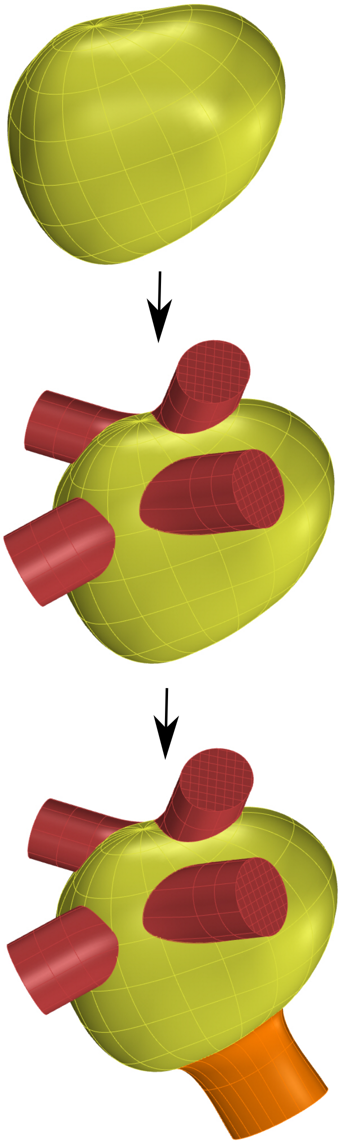

Figure S1. Construction workflow of the atrium model. From top to bottom: main ideal shape, pulmonary veins and mitral valve.

2 LAA WALL MOTION: FUNCTIONS DEFINITION

The displacement functions $F_L(t)$ and $F_M(t)$ applied to the lateral and medial walls of the distal LAA portion are obtained as sum of two contributes, corresponding to the two terms in eq. S1 and in the eq. S2.

- The first term corresponds to the translation motion of the appendage distal part, and corresponds to the mean value of displacement between the lateral and medial walls. In detail, it is zero at the proximal-distal junction and linearly increases towards the appendage tip, where reaches the maximum.
- The second term corresponds to the enlargement or the shrinkage of the appendage, due to the differential motion of the lateral and medial walls. It corresponds to the product of two factors: the displacement departure from the average, and a parabolic distribution with vertex of unit value (namely

h

Table S1. Clinical measures used to build the standard atrium shape. LS: left superior vein; LI: left inferior vein; RS: right superior vein; RI: right inferior vein; MV: mitral valve; PV: pulmonary veins

	Length (mm)
Planar dimensions	
X	76
Y	56
Z	63
Orifice dimensions (DminXDmax)	
LS	15x20
LI	14x18
RS	17x22
RI	17x20
Distances PV	
LS-LI	33
RS-RI	34
LS-RS	33
LI-RI	39
Distances PV-MV	
LS-MV	73
RS-MV	69
LI-MV	46
RI-MV	51

h

Table S2. Parameter values and geometrical aspects for the integration of the atrium and the appendage into a unique idealized model, for the cases *Healthy* and *Pathological*.

	SR	AF
Distance MV-LAA (mm)	28.46	31.74
Distance LSPV-LAA (mm)	14.23	15.87
Tip orientation	Downward	Downward

$\delta_L(z)$ and $\delta_M(z)$) and zero value at the tip and at the proximal-distal junction. The parabola reaches the maximum value at the center of the LAA distal part.

$F_L(t)$ and $F_M(t)$ were applied to the mesh nodes of the LAA surface along the y direction (see Figure S3), and are defined as follow:

$$F_L(z, x_0, y_0, z_0, t) = \Delta_{L,M} * \frac{z_0}{L_d} + (T_L - \Delta_{L,M}) * \delta_L(z) \tag{S1}$$

$$F_M(z, x_0, y_0, z_0, t) = \Delta_{L,M} * \frac{z_0 + y_0 \cot\theta}{L_d + y_0 \cot\theta} + (T_M - \Delta_{L,M}) * \delta_M(z) \tag{S2}$$

where x_0, y_0, z_0 are the initial coordinates of the mesh points, z is the coordinate of the points at time t, $\Delta_{L,M} = Mean(s_L(t), s_M(t))$, L_d is the axis length of the distal part of the appendage, θ is the angle between the distal-proximal junction and the distal axis (see Figure S3). While, the functions $T_L(t)$ and $T_M(t)$ are defined as follow:

$$T_L(x_0, y_0, t) = s_L(t) * \sin(\arctan(y_0, x_0))^2 + \Delta_{L,M} * (\cos(\arctan(y_0, x_0))^2 \tag{S3}$$

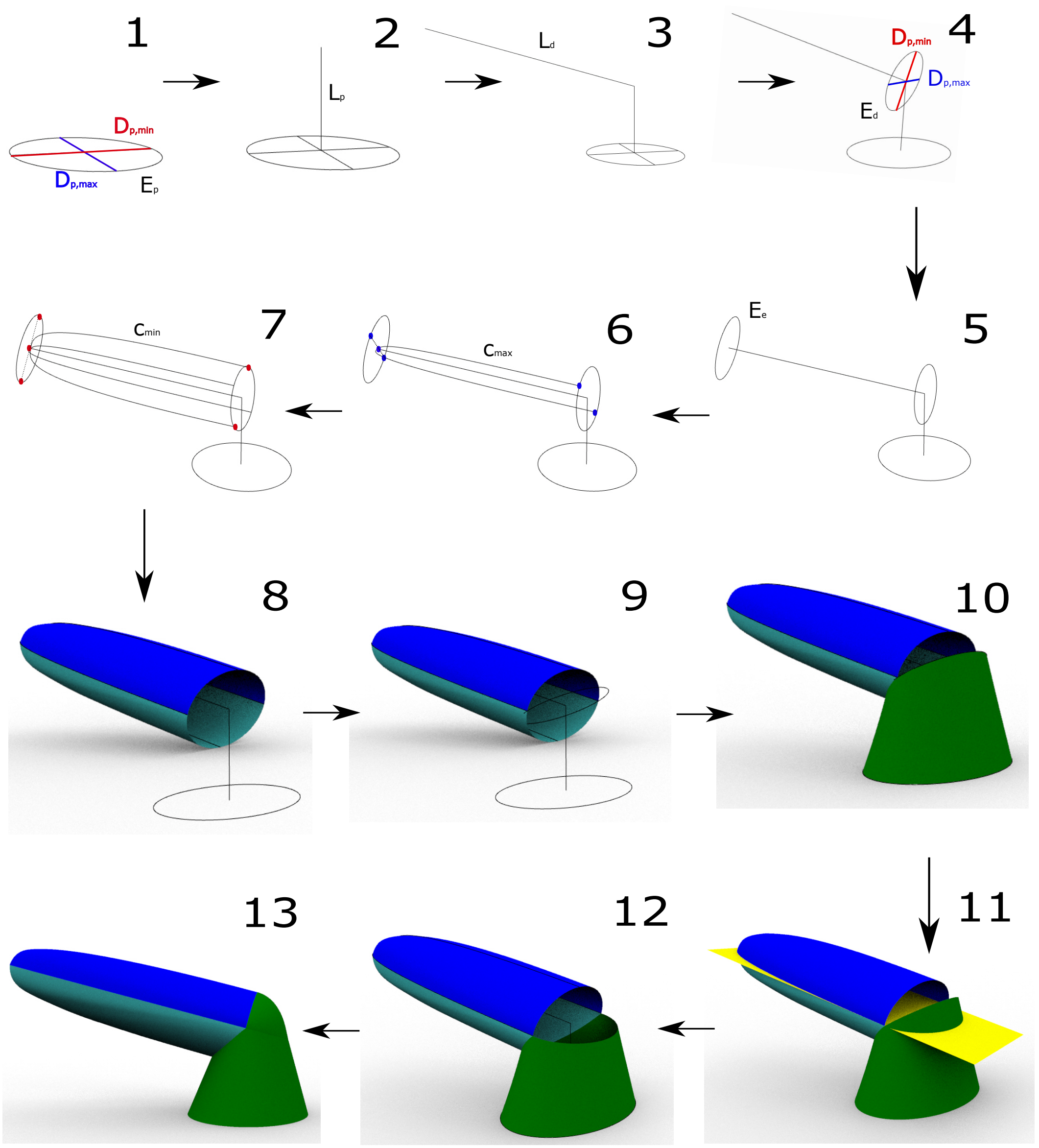

Figure S2. Construction workflow of the appendage model.

$$T_M(x_0, y_0, t) = s_M(t) * \sin(\arctan(y_0, x_0))^2 + \Delta_{L,M} * (\cos(\arctan(y_0, x_0))^2 \quad \text{(S4)}$$

These two functions were designed to fit $s_L(t)$ and $s_M(t)$ along the traversal profile of the appendage, corresponding to the circumferential direction. Each function assumes the maximum value at the wall center (at 90° of the transverse circumference) and the mean value $\Delta_{L,M}$ at the two extremes, 0 and 180°.

The same displacement functions $F_L(t)$ and $F_M(t)$ (eq. S1, eq. S2) are applied for the two cases *Healthy* and *Pathological*, changing the parameter values of s_L, s_M, L_d and θ. The functions s_L and s_M are described in the main text (Section 2.2) for the two cases studied. While, for the *Healthy* case $L_d = 31\ mm$, $\theta = 57°$ and for the *Pathological* case $L_d = 34\ mm$, $\theta = 50°$.

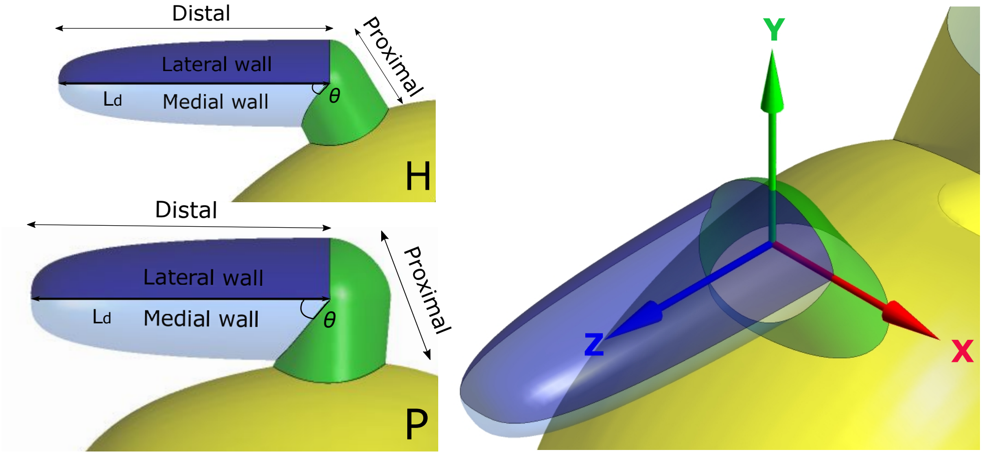

Figure S3. In the left panel, the main parts of the appendage: proximal and distal, lateral and medial wall, distal axis of length L_d and θ angle at the distal-proximal junction, for the Healthy (H) and Pathological (P) cases. In the right panel, the reference Cartesian axes. The axes were choose such that the z axis is directed along the longitudinal axis of the appendage distal part, the y axis is directed towards the lateral part and the x axis towards the junction between lateral and medial parts.

3 ATRIUM WALL VELOCITY

To define the curve of LA volume variation, three volume values were used, corresponding to three phases of the cardiac cycle:

- Maximum Volume (V_{max}), prior to mitral valve opening;
- Pre-Contraction Volume (V_{preA}), prior to *A wave*, corresponding to atrial contraction;
- Minimum Volume (V_{min}), at the end of atrial contraction.

Since the two atrium geometries, healthy and pathological, are coherent with the atrium volumes measured in two subject groups (see Section 2.1.2), the same volumes were used to reproduce the atrial contraction for the two cases, *Healthy* and *Pathological* (Lacomis et al., 2007). For the *Hybrid* model, based on the healthy geometry and pathological contraction pattern, the three values were computed setting two conditions: the average of V_{max} and V_{min} as in the *Healthy* case and the ratios $\frac{V_{preA}}{V_{max}}$ and $\frac{V_{preA}}{V_{min}}$ as in the *Pathological* case. Using these values, a volume-time function was computed as sum of sine curves for each case (see Figure S4 in top panel). The velocity of the atrium wall was obtained from the volume-time function, first normalizing respect to the area of the atrium surface, then deriving respect to the time (see Figure S4 in the bottom panel). Of note, in the *Pathological* and *Hybrid* models the curve amplitude reduces during the mitral valve closing and the atrial contraction.

4 PRESSURE

The pressure curves were obtained as result of four CFD simulations (one for each studied case), with different flowrates applied to the mitral valve outlet. Here a uniformly distributed velocity, varying in time in order to provide the flowrate for healthy and AF conditions, was set. In detail, two different velocity curves were used, first one represents the flowrate in Sinus Rhythm and second one represents the flowrate in atrial fibrillation, obtained removing the *A wave* associated with the atrial contraction (see Figure 4 left panels in the main text). The curve was normalized respect to the mitral orifice area, respectively $2.68\ cm^2$

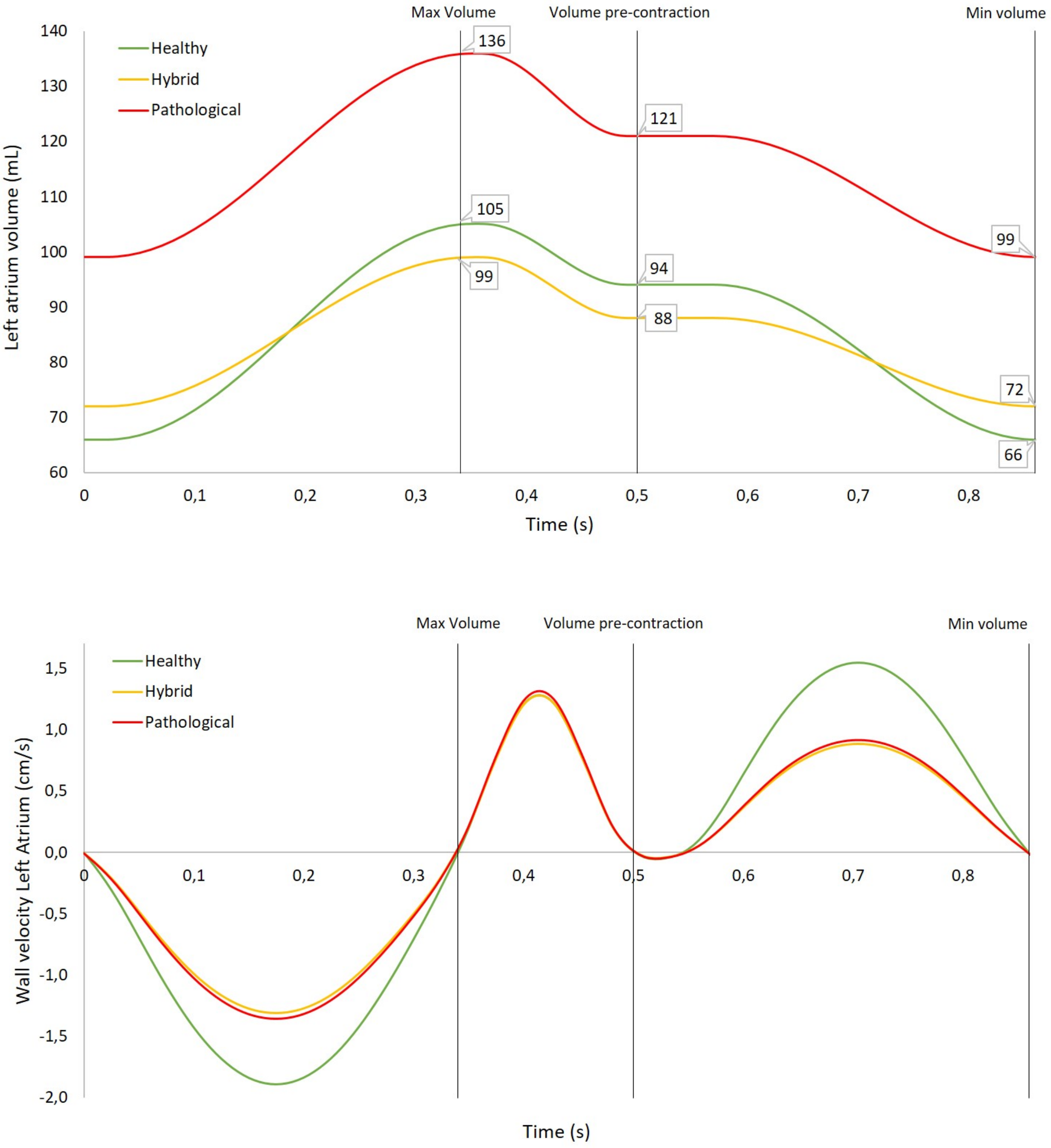

Figure S4. Top panel: volume variation of the left atrium during a cardiac cycle; each curve is based on three main values: Maximum Volume, Pre-contraction Volume and Minimum Volume. Bottom panel: velocity of atrium wall during a cardiac cycle, corresponding to the derivative of the volume curve in the top panel.

for the cases *Healthy*, *Rigid* and *Hybrid*, and $3.54\ cm^2$ for the *Pathological* case. Three cardiac cycles were simulated and the average pressure in time at the outlet area was computed (see Figure 4 panels B and D in the main text).

REFERENCES

Bloodworth, C. H., Pierce, E. L., Easley, T. F., Drach, A., Khalighi, A. H., Toma, M., et al. (2017). Ex Vivo Methods for Informing Computational Models of the Mitral Valve. *Annals of Biomedical Engineering*

doi:10.1007/s10439-016-1734-z

Budge, L. P., Shaffer, K. M., Moorman, J. R., Lake, D. E., Ferguson, J. D., and Mangrum, J. M. (2008). Analysis of in vivo left atrial appendage morphology in patients with atrial fibrillation: A direct comparison of transesophageal echocardiography, planar cardiac CT, and segmented three-dimensional cardiac CT. *Journal of Interventional Cardiac Electrophysiology* 23, 87–93. doi:10.1007/s10840-008-9281-7

Lacomis, J. M., Goitein, O., Deible, C., Moran, P. L., Mamone, G., Madan, S., et al. (2007). Dynamic multidimensional imaging of the human left atrial appendage. *Europace* 9, 1134–1140. doi:10.1093/europace/eum227

Li, C. Y., Gao, B. L., Liu, X. W., Fan, Q. Y., Zhang, X. J., Liu, G. C., et al. (2015). Quantitative evaluation of the substantially variable morphology and function of the left atrial appendage and its relation with adjacent structures. *PLoS ONE* 10. doi:10.1371/journal.pone.0126818

Lim, K. H., Yeo, J. H., and Duran, C. (2005). Three-dimensional asymmetrical modeling of the mitral valve: a finite element study with dynamic boundaries. *J Heart Valve Dis* 14, 386–392

Mori, M., Kanzaki, H., Amaki, M., Ohara, T., Hasegawa, T., Takahama, H., et al. (2011). Impact of reduced left atrial functions on diagnosis of paroxysmal atrial fibrillation: results from analysis of time-left atrial volume curve determined by two-dimensional speckle tracking. *Journal of cardiology* 57, 89–94

Nattel, S. and Harada, M. (2014). Atrial remodeling and atrial fibrillation: recent advances and translational perspectives. *Journal of the American College of Cardiology* 63, 2335–2345

Sanfilippo, A. J., Abascal, V. M., Sheehan, M., Oertel, L. B., Harrigan, P., Hughes, R. A., et al. (1990). Atrial enlargement as a consequence of atrial fibrillation. a prospective echocardiographic study. *Circulation* 82, 792–797

Schwartzman, D., Lacomis, J., and Wigginton, W. G. (2003). Characterization of left atrium and distal pulmonary vein morphology using multidimensional computed tomography. *Journal of the American College of Cardiology* doi:10.1016/S0735-1097(03)00124-4

Varela, M., Bisbal, F., Zacur, E., Berruezo, A., Aslanidi, O. V., Mont, L., et al. (2017). Novel computational analysis of left atrial anatomy improves prediction of atrial fibrillation recurrence after ablation. *Frontiers in physiology* 8, 68
